# Advanced Needle-Based Strategies for Complex Calcified Peripheral Artery Disease: A Systematic Review

**DOI:** 10.1016/j.jscai.2026.105306

**Published:** 2026-04-09

**Authors:** Rendra Mahardhika Putra, Suci Indriani, Johanes Nugroho Eko Putranto, Raden Mohammad Budiarto, Nadya Luthfah, Kevin Francio, Wynne Widiarti, Melly Susanti, Seung Woon Rha

**Affiliations:** a Department of Cardiology and Vascular Medicine, Faculty of Medicine, Universitas Airlangga, Surabaya, Indonesia; b Department of Cardiology and Vascular Medicine, Universitas Airlangga Hospital, Surabaya, Indonesia; c Department of Cardiology and Vascular Medicine, Faculty of Medicine, Universitas Indonesia/National Cardiovascular Center, Harapan Kita Hospital, Jakarta, Indonesia; d Department of Cardiology and Vascular Medicine, Dr. Soetomo General Academic Hospital, Surabaya, Indonesia; e Faculty of Medicine, Universitas Airlangga, Surabaya, Indonesia; f Ministry of Health Central General Hospital, Surabaya, Indonesia; g Cardiovascular Center, Korea University Guro Hospital, Seoul, South Korea

**Keywords:** angioplasty, complex lesions, endovascular interventions, peripheral arterial disease, vascular calcification

## Abstract

**Background:**

Severely calcified peripheral artery disease (PAD) lesions that cannot be fully dilated by conventional balloon angioplasty pose major therapeutic challenges, especially when atherectomy devices are unavailable. Needle-based, plaque-modifying strategies have emerged as adjunctive or bailout approaches to facilitate lesion crossing and vessel expansion in such complex settings.

**Methods:**

A systematic review was conducted in accordance with Preferred Reporting Items for Systematic Reviews and Meta-Analyses (PRISMA) 2020 guidelines. Scientific databases were searched for studies evaluating needle-based interventions for heavily calcified PAD.

**Results:**

Fifteen studies encompassing 101 patients were included, involving techniques such as fracking (hydraulic plaque cracking), percutaneous direct needle puncture of calcified plaque (PIERCE), BAMBOO SPEAR (needle drilling method), biopsy forceps plaque extraction, and FRAP-CROSS technique. Lesions were located in the common femoral, superficial femoral, anterior tibial, and tarsal arteries. Technical success ranged from 94% to 100% across reported studies. Fracking achieved substantial minimal luminal area gains (6-10 mm^2^ to >22 mm^2^), corresponding with 90% 12-month patency and 94% target lesion revascularization–free survival. PIERCE and its modifications enabled safe advancement through calcified segments refractory to conventional crossing, with minimal complications. BAMBOO SPEAR and biopsy forceps extraction provided effective plaque debulking, complete stent expansion, and 100% procedural success in reported cases. FRAP-CROSS, combining fracking and Rendezvous-PIERCE, safely facilitated wiring across calcified femoropopliteal occlusions. Adverse limb events were rare, and long-term durability appeared comparable to atherectomy-based outcomes.

**Conclusions:**

Needle-based, plaque-modifying strategies appear feasible for complex calcified PAD, particularly in resource-limited settings. Further prospective studies are warranted to define optimal indications, standardize techniques, and validate long-term outcomes.

## Introduction

Peripheral artery disease (PAD) remains a significant global health concern, especially in patients presenting with complex and/or heavily calcified lesions. Calcified plaques further complicate the scenario by creating a rigid, noncompliant arterial lesion, impeding blood flow, and elevating the risk of limb-threatening ischemia. Together, complex and heavily calcified lesions contribute to poor procedural outcomes and present a major barrier for successful endovascular revascularization.^1^^,^^2^ Treating these complex lesions remains technically challenging. Standard endovascular techniques, including guide wire crossing, balloon angioplasty, drug-coated balloons, and atherectomy, frequently fail in the presence of dense, concentric, or nodular heavy calcium. Calcification can block guide wire passage, prevent device advancement, and resist balloon expansion, often leading to procedural failure, suboptimal procedural outcomes, or the need for surgical bypass.^3^ Moreover, atherectomy devices, although helpful in modifying superficial calcium, carry risks such as vessel perforation and distal embolization, and are less effective in targeting deep calcium. These limitations have led to the development of alternative, mechanically focused, cost-effective solutions.^4^

Needle-based recanalization techniques have been developed to overcome the structural resistance of heavily calcified lesions. Among these, fracking (hydraulic plaque cracking) and percutaneous direct needle puncture of calcified plaque (PIERCE) are the most widely reported. Fracking involves delivering hydraulic force through a percutaneously inserted needle during balloon inflation, producing controlled microfractures in deep calcium and enabling vessel expansion.^5^ In contrast, PIERCE uses a fine specialized needle to puncture or crack heavily calcified occlusions, creating a channel for guide wire passage where standard approaches fail. Variations, including wall PIERCE, inner PIERCE, and rendezvous-PIERCE, have further broadened applicability to infrainguinal and distal territories.^6^ Both techniques are especially valuable when conventional devices cannot cross or adequately modify the lesion, offering novel alternatives for revascularization. These techniques are particularly useful in focal, dense, and deep calcified lesions where atherectomy devices are not effective. Although evidence for these remains limited to case reports and series, they underscore the versatility of simple, inexpensive tools in anatomically hostile lesions. This review aims to evaluate and compare the clinical utility of various needle-based and plaque-modifying techniques for complex calcified peripheral artery occlusions. By synthesizing data from recent studies, we examine their feasibility, technical outcomes, safety, and potential advantages over existing approaches.

## Materials and methods

This systematic review was conducted in accordance with the 2020 Preferred Reporting Items for Systematic Reviews and Meta-Analyses (PRISMA) guidelines (Supplemental Table S1 and Supplemental Figure S1).^7^ The protocol was prospectively registered with PROSPERO under the identifier CRD420251113480.

### Study eligibility criteria

To comprehensively capture the most current evidence on various needle-based and plaque-modifying techniques for complex calcified PAD, certain inclusion criteria were applied. Studies were considered eligible if they met the following parameters: (1) involved adult patients (≥18 years) undergoing endovascular intervention for complex and/or heavily calcified PAD; (2) evaluated needle-based strategies such as the PIERCE technique, fracking technique, or other plaque-modifying and calcium-disruption methods, with or without comparison to standard crossing approaches; and (3) were published in English. All study designs were included. There were no restrictions placed on the year of publication to ensure comprehensive capture of evolving device technology and procedural strategies. Studies focusing solely on coronary interventions, nonhuman subjects, or lacking accessible full text articles were excluded.

### Literature search and study selection

An extensive literature search was performed across scientific databases, including PubMed, Web of Science, ScienceDirect, Springer, Cochrane Library, ClinicalTrials.gov, and ProQuest up to July 31, 2025. The search strategy combined keywords and Boolean operators “Peripheral Artery Disease,” OR “Peripheral Arterial Occlusion,” AND “Complex Lesions,” OR “Calcified Lesions,” OR “Vascular Calcification,” AND “Crossing Techniques,” OR “Subintimal Angioplasty,” OR “Needle-Based Techniques,” OR “Plaque Modification,” OR “Plaque-Modifying Devices,” OR “Fracking,” OR “PIERCE Technique.” Duplicate records were removed prior to the screening phase. Two independent reviewers screened titles and abstracts, followed by full text evaluation for inclusion. Additional studies were identified through backward citation tracking and gray literature screening.

### Data extraction process

Two reviewers independently extracted data using a standardized approach. In cases of missing or incomplete information, study authors were contacted for clarification. Extracted data are consolidated in Supplemental Tables S2 and S3. Discrepancies during the extraction process were resolved through consensus with a third reviewer.

### Quality assessment

To assess the methodological quality of observational studies, the Newcastle-Ottawa Scale was employed.^8^ The Newcastle-Ottawa Scale evaluates studies based on selection of study groups, group comparability, and outcome or exposure assessment, with a maximum score of 9 points. For case reports and case series, the Joanna Briggs Institute critical appraisal tools were utilized.^9^ Quality assessment was conducted independently by 2 reviewers, with disagreements resolved by a third reviewer. No studies were found to have a high risk of bias (Supplemental Tables S4 and S5).

### Data synthesis

Because of the heterogeneity of study designs and reported outcomes, a meta-analytic approach was not feasible. As a result, data were synthesized through qualitative analysis to summarize the available evidence.

## Results

### Baseline characteristics

A total of 15 studies involving 101 patients were included in this review.^5^^,^^6^^,^^10^, ^11^, ^12^, ^13^, ^14^, ^15^, ^16^, ^17^, ^18^, ^19^, ^20^, ^21^, ^22^ Study designs consisted of 9 case reports, 4 case series, and 2 retrospective cohort studies. Most studies were conducted in Japan, with additional contributions from China, Singapore, Thailand, Italy, the United Kingdom, and the United Arab Emirates. The majority of the patients were male, with proportions ranging from 25% to 100%. Patient ages spanned a wide range, from 55 to 89 years, with most studies involving older adults, typically over 65 years old. The body mass index (BMI) was variably reported; in 1 comparative study by Haraguchi et al^10^, the mean BMI was 21.30 ± 2.90 kg/m^2^ in the fracking group and 22.80 ± 4.20 kg/m^2^ in the balloon angioplasty group, whereas other reports noted either “obese” or did not specify BMI at all.

Comorbidities were frequently present. Hypertension was the most consistently reported, with a prevalence ranging from 73.30% to 100% among those who reported this metric. Diabetes mellitus was present in up to 76% of patients, particularly in retrospective cohort data and fracking cases. Dyslipidemia and a history of smoking were also prevalent in several series, but were often not explicitly reported in case reports. Coronary artery disease was documented in several patients, with notable mentions of individuals post–coronary artery bypass graft in Nakama et al^19^ and Troisi et al. Chronic kidney disease was observed in up to 100% of patients in certain cohorts, with 1 report^20^ involving a posttransplant case.

### Procedural and lesion characteristics

**Figure 1:**
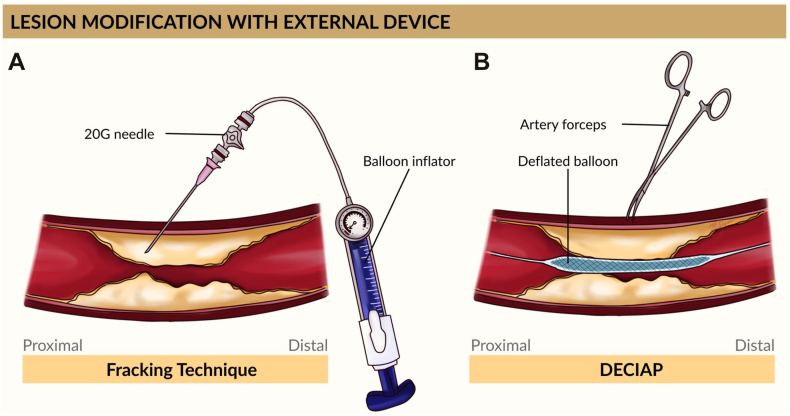
**Lesion modificationusing external devices.(A)** Fracking technique using hydraulic pressure to crack deep calcified plaque. **(B)** DECIAP technique using artery forceps to crush calcified plaque. DECIAP, direct endarterectomy by clamp-induced artery plaque cracking.

Across the included studies, needle-based recanalization techniques were most frequently applied in the common femoral artery (CFA),^5^^,^^10^^,^^13^ followed by PIERCE-based techniques and their modifications applied across a broad anatomic spectrum, including the superficial femoral artery (SFA), and the iliac, femoropopliteal, tibial, and pedal arteries. These techniques were predominantly used to facilitate wire passage or enlarge heavily calcified lumens, particularly after failure of conventional guide wire or balloon crossing attempts.^5^^,^^6^^,^^10^, ^11^, ^12^, ^13^, ^14^, ^15^, ^16^, ^17^, ^18^, ^19^, ^20^, ^21^, ^22^ The fracking technique was reported by Haraguchi et al^5^ and Haraguchi et al^10^. In Haraguchi et al^5^, 2 cases involved severely eccentric calcified plaques in the mid-to-distal CFA, with baseline minimal lumen areas (MLA) of 6.20 mm^2^ and 10 mm^2^, respectively, and baseline stenoses of 94% to 96%. The procedure entailed delivering hydraulic pressure via an 18-gauge needle (Terumo) during balloon inflation until a pressure drop signaled successful calcium fracture (Figure 1A). Haraguchi et al^10^ included a retrospective cohort of 59 patients and compared fracking with balloon angioplasty for CFA calcification. In this study, fracking-treated lesions demonstrated more extensive 180° to 360° calcification (81.30% vs 48.60% in the balloon group), greater acute luminal gain (16.40 ± 4.80 mm^2^ vs 6.80 ± 4.40 mm^2^), and lower residual plaque burden (49% vs 73%). Procedural times averaged 10.10 ± 3.80 minutes, with radiation times of 25.50 ± 13.80 minutes (fracking) vs 51.60 ± 44.30 minutes (balloon). This study also documented that the mean procedure time for fracking was 10.10 ± 3.80 minutes, with substantially shorter radiation exposure times (25.50 ± 13.80 minutes) compared with balloon angioplasty, which demonstrated markedly longer radiation times (51.60 ± 44.30 minutes).^10^

**Figure 2:**
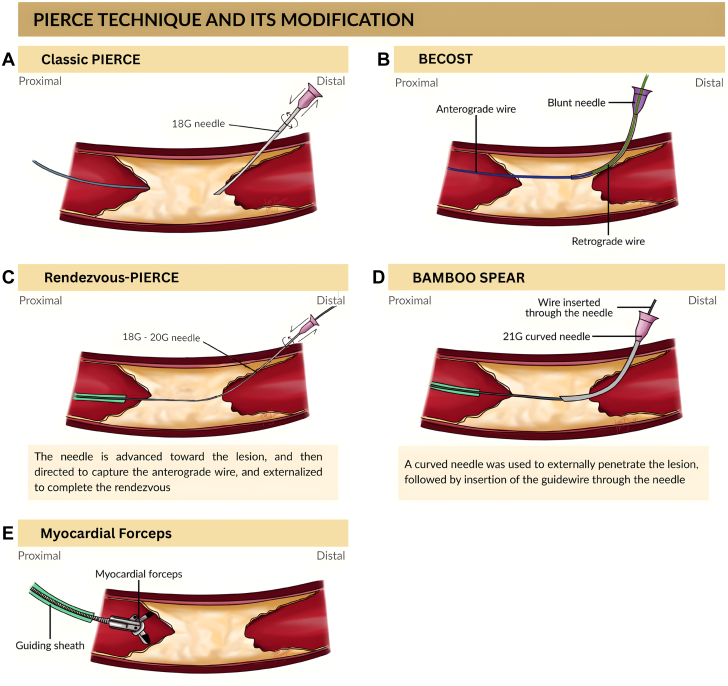
**Percutaneous direct needle puncture of calcified plaque** (**PIERCE) technique and its modifications.(A) Direct PIERCE technique using percutaneous needle puncture of calcified plaque to facilitate balloon or wire passage. (B) BECOST technique using a blunt-tip needle over a strained through-and-through wire to crack calcified lesions. (C) Rendezvous-PIERCE technique using retrograde needle drilling toward an antegrade wire through severe calcification. (D) BAMBOO SPEAR technique with curved bare-metal needle drilling and adjunct balloon angioplasty. (E) Biopsy forceps technique for mechanical excision of calcified plaque. BAMBOO SPEAR, needle drilling method; BECOST, blunt endoluminal cracking over strained through-wire.**

Needle puncture methods and PIERCE technique modifications were described in multiple reports (Figure 2). Haraguchi et al^11^ applied a Rendezvous-PIERCE modification in calcified anterior tibial and femoropopliteal lesions: an antegrade and retrograde wire were aligned, and a retrograde 18- to 20-G needle (outer diameters 1.30 mm and 0.90 mm) drilled through the calcified segment toward the antegrade wire without externalization (Figure 2C). Ichihashi et al^6^ described 4 cases (3 SFA, 1 posterior tibial artery [PTA]) using direct PIERCE puncture with a 19-G needle (HAKKO ELASTER, Hakko) in 2 cases and a 16-G needle in 2 others (Figure 2A). Takamura et al^20^ used a 21-G needle (HAKKO ELASTER) for a heavily calcified lesion in the distal SFA. Takei et al^21^ treated severe below-the-ankle calcification (anterior tibial artery [ATA], PTA, dorsal artery) with inner PIERCE, guiding a 20-G, 105 mm puncture needle (MEDIKIT) or 20-G, 310 mm biopsy needle (NIPRO) along an externalized wire. Nakama et al^19^ employed an 18-G, 20 cm PTCD needle (Happy-cath, Medikit) for distal PTA and lateral plantar artery lesions. Troisi et al^22^ used a 20-G radial cannulation needle for medial tarsal artery calcification to enable angiosome-targeted revascularization. In addition, Haraguchi et al^12^ described a hybrid strategy termed FRAP-CROSS, which combined fracking with Rendezvous-PIERCE to facilitate intracalcium guide wire passage in femoropopliteal diffuse calcified occlusions. In this report, the combined approach successfully achieved wiring across the occlusion after failure of standard guide wire, catheter, and balloon advancement techniques. This hybrid method capitalizes on hydraulic calcium fracturing to weaken rigid plaque before needle rendezvous, thereby enabling safe and controlled guide wire advancement without the need for subintimal tracking or stenting.

Several unique single-technique reports were included. For example, Hayakawa et al^13^ developed a needle drilling technique called “direct BAre Metal needle puncture and BallOOn angioplaSty in calcified PlaquEs of the common femoral ARtery guided by angiography” (needle drilling method [BAMBOO SPEAR]) (Figure 2D). Hirano et al^14^ used biopsy forceps to physically excise severely calcified plaque at the SFA ostium, performing 21 extractions to form a stentable lumen (Figure 2E). Horsirimanont and Kittitirapong^15^ described blunt endoluminal cracking over strained through-wire (BECOST) for heavily calcified below-the-knee (BTK) lesions: a blunt-tip needle was advanced over a through-and-through-wire to crack the calcified segment, with procedure times of 230 to 510 minutes (Figure 2B). Huang et al^16^ performed sharp recanalization using the stiff end of a Terumo Radifocus glidewire supported by a 5F Judkins Right catheter to penetrate calcified caps in SFA/popliteal lesions. Kim et al^17^ applied the same concept to calcified lesions in the common iliac artery (CIA) and ATA. Kum et al^18^ described direct endarterectomy by clamp-induced artery plaque cracking (DECIAP) after failed PIERCE; a 1 cm skin incision was made over the lesion, and 16-G artery forceps were inserted to crush calcified wall segments under fluoroscopic guidance (Figure 1B). Details about all included needle-based or plaque-modifying techniques are summarized in Table 1.^5^^,^^6^^,^^10^, ^11^, ^12^, ^13^, ^14^, ^15^, ^16^, ^17^, ^18^, ^19^, ^20^, ^21^, ^22^

**Table 1:** Summary of included studies on advanced needle-based recanalization techniques in complex calcified PAD

Reference, year	Technique	Lesion location	Identified lesion	Needle/device	Patients (n)	Key outcomes	Complications
Haraguchi et al,^5^ 2021	Fracking	CFA	Calcified lesion with suboptimal post-balloon MLA documented by IVUS with cutting balloon and noncompliant balloon	18-G needle	2	MLA ↑ from ∼7→27 mm^2^; 100% success; patency >24 mo	Immediate evaluation after the procedure documented no complications
Haraguchi et al,^10^ 2023	Fracking vs balloon	CFA	Calcified lesions with residual angiographic stenosis of >50% or inadequate lumen area after balloon angioplasty, estimated by IVUS	18-G needle	59	Success 97% vs 74%; 1-y patency 90% vs 49%; lower restenosis	Immediate evaluation after the procedure documented no complications at the target lesions. 2/32 (6.25%) cases of hemorrhage at the sheath insertion site were noted in the fracking group
Haraguchi et al,^11^ 2024	Rendezvous-PIERCE	ATA, FP	Calcified lesions that were uncrossable with microcatheters and balloons	18-20 G retrograde needle	2	Balloon crossed, hemostasis	Immediate evaluation after the procedure documented no complications at the target lesions.
Haraguchi et al,^12^ 2025	FRAP-CROSS	Femoropopliteal	Diffuse calcified lesions that were uncrossable with the highest tip-loaded guide wire	20-G metal needle without the plastic outer sheath from an 18-G needle (Terumo) or a 21-G metal needle (micro-puncture introducer set, Cook Medical)	1	Successful guide wire advancement and subsequent balloon angioplasty, supporting stentless treatment	Immediate evaluation after the procedure documented no perforation, no distal embolization, and no flow-limiting dissection
Hayakawa et al,^13^ 2022	BAMBOO SPEAR	CFA	Heavily calcified lesion with total occlusion	21-G curved needle	1	Vessel patency at 5 mo	The possibility of distal embolism cannot be ruled out. Therefore, a filter wire (Parachute filter wire; Keisei Medical) was placed in the popliteal artery
Hirano et al,^14^ 2021	Biopsy forceps	SFA ostium	Heavily calcified SFA plaque protruded into CFA, confirmed with IVUS; risk of stenting underexpansion and potential plaque shift toward the DFA	Myocardial biopsy forceps	1	ABI normalized; no restenosis at 9 mo	Not reported
Horsirimanont and Kittitirapong,^15^ 2025	BECOST	BTK	Calcified lesions that were uncrossable with the balloon Armada XT 2/20 balloon catheter (Abbott Vascular International BVBA)	Blunt needle	4	100% success; 75% wound healing	Immediate evaluation after the procedure documented 3 cases (75%) of nonflow-limited dissection; 1 case (25%) required balloon-assisted thrombin injection for puncture-site hemorrhage
Huang et al,^16^ 2012	Sharp recanalization	SFA, popliteal	Calcified lesions that were uncrossable with 0.014-in Conquest pro CTO guide wire (Asahi Intec), 0.018-in V18-control wire (Boston Scientific), or 0.035-in Terumo stiff glidewire (Radifocus, Terumo), along with the balloon or exchange catheter	Stiff Terumo wire	2	ABI improved; 1 toe amputation; 1 reintervention	1 (25%) case of reintervention 7 mo afterward because of additional lesion in the left CIA and diffuse ISR in the SFA
Ichihashi et al,^6^ 2014	PIERCE	SFA, PTA	Calcified lesions that were uncrossable. In cases 1, 2, and 4, the guide wire crossed, but neither support catheters nor a 1.5-mm balloon could advance because of heavy calcification. In cases 3 and 4, the calcified lesions were resistant to balloon dilation even at pressures up to 27 atm	16- to 19-G needle	4	Balloon crossing in all; 3 stented	Immediate evaluation after the procedure documented 2 (50%) minor hemorrhage from the punctured tract
Kim et al,^17^ 2021	Sharp recanalization	CIA, ATA	Calcified lesions that were uncrossable with the conventional catheter and guide wire technique	Stiff wire	2	Successful lesion crossing	Not reported
Kum et al,^18^ 2020	DECIAP	Various BTK	Calcified lesions that were uncrossable or recoiled lesions that could not be treated by high-pressure noncompliant balloon	16-G artery forceps	4	Balloon success in all; ≤30% residual stenosis (2)	Immediate evaluation after the procedure documented 1 (25%) case of perforation in the related artery where the technique was performed; 1 (25%) patient died 6 d after the procedure
Nakama et al,^19^ 2020	Inner PIERCE	Distal PTA, plantar	Calcified lesions that were crossable with a guide wire, but other devices could not pass through	18-G PTCD	1	Wound healed at 4 mo; patency at 1 y	Immediate evaluation after the procedure documented no complication
Takamura et al,^20^ 2016	PIERCE	Distal SFA	Calcified lesions that were uncrossable with a guide wire	21-G needle	1	Optimal stent expansion	Not reported
Takei et al,^21^ 2021	Inner PIERCE	BTK, ankle arteries	Calcified lesions that were uncrossable with low-profile balloons, and balloon indentation persisted even when inflated to pressures above the rated burst level	20-G puncture/biopsy needle	18	94% success; <30% residual stenosis; no complications	Immediate evaluation after the procedure documented no complication
Troisi et al,^22^ 2023	PIERCE	Medial tarsal artery	Under fluoroscopic imaging, the medial tarsal artery was found to have severe calcification, preventing antegrade recanalization with multiple 0.014-inch guide wires. Ultrasound guidance to directly puncture the medial tarsal artery retrograde with a 20-gauge needle. After 5 attempts, a 0.014-inch guide wire was successfully advanced retrograde into the true-lumen and into the peroneal artery	20-G radial cannula	1	Direct flow; wound healing in 10 d	Not reported

ABI, ankle-brachial index; ATA, anterior tibial artery; BAMBOO SPEAR, needle drilling method; BECOST, blunt endoluminal cracking over strained through-wire; BTK, below-the-knee; CFA, common femoral artery; CIA, common iliac artery; DECIAP, direct endarterectomy by clamp-induced artery plaque cracking; DFA, deep femoral artery; FP, femoro-popliteal; ISR, in-stent restenosis; IVUS, intravascular ultrasound; MLA, minimal lumen area; PAD, peripheral artery disease; PIERCE, percutaneous direct needle puncture of calcified plaque; PTA, posterior tibial artery; PTCD, percutaneous transhepatic cholangiographic drainage; SFA, superficial femoral artery.

### Safety, clinical, and procedural outcomes

Haraguchi et al^5^ reported 100% technical success with fracking, increasing MLA from ∼7 mm^2^ to >27 mm^2^, 0% residual stenosis, and patency >24 months with no reinterventions or complications. Haraguchi et al^10^ demonstrated procedural success of 96.90% with fracking vs 74.30% for balloon angioplasty, postprocedural MLA of 22.10 ± 4 mm^2^ vs 12.70 ± 3.30 mm^2^, 1-year primary patency of 89.80% vs 49.20%, restenosis rates of 9.40% vs 42.80%, reocclusion rates of 0% vs 5.70%, and superior freedom from target lesion revascularization (93.5% vs 74.20%) and major adverse limb event (76.90% vs 48.60%). No major complications occurred.

For PIERCE and its variants, Haraguchi et al^11^ reported no complications, sustained asymptomatic status at 6 to 12 months, and no reinterventions. Ichihashi et al^6^ achieved balloon crossing in all cases, with 3 stented successfully; 2 patients experienced minor tract bleeding but no distal embolization or perioperative adverse events. Takamura et al^20^ achieved optimal stent expansion without adverse events. Takei et al^21^ reported 94.40% technical success, <30% residual stenosis in all cases, no complications, and 100% device delivery. Nakama et al^19^ achieved wound healing by 4 months, with no restenosis at 1 year. Troisi et al^22^ restored direct flow with duplex-confirmed patency (toe-brachial index 0.92) and intraoperative transcutaneous oximetry improvement of +7%, leading to near-complete wound healing within 10 days. Haraguchi et al^12^ (FRAP-CROSS) demonstrated successful guide wire advancement through heavily calcified femoropopliteal occlusions without procedural complications. The technique enabled subsequent balloon angioplasty and stentless treatment, with no flow-limiting dissection, no perforation, and no distal embolization reported on immediate post-procedural evaluation.

In addition, Hayakawa et al^13^ (BAMBOO SPEAR) achieved vessel patency at 5 months with no complications. Hirano et al^14^ (biopsy forceps extraction) achieved a normalized ankle-brachial index (1.06) and no restenosis at 9 months. Horsirimanont and Kittitirapong^15^ (BECOST) reported 100% technical success, minimal nonflow-limiting dissections in 75%, 1 stent implantation, 1 puncture-site complication managed with thrombin injection, 75% complete wound healing within 3 months, and 1 reintervention for restenosis. Huang et al^16^ (sharp recanalization) improved ankle-brachial index to 0.78 and 0.94, with 1 toe amputation, 1 CIA reintervention at 7 months, and no acute ischemic events. Kim et al^17^ achieved successes in advancing heavily calcified lesions in both CIA and ATA without complications. Kum et al^18^ (DECIAP) reported balloon success in all 4 cases, ≤30% residual stenosis in half, 1 arterial perforation managed by balloon inflation, 1 death from unrelated sepsis, and 1 reintervention at 3 months.

Procedural success was generally favorable, though several perioperative (<30 days) complications were reported.^6^^,^^10^^,^^15^^,^^16^^,^^18^ These included nonflow-limiting dissections, minor hemorrhage along puncture tracts, arterial perforations, and delayed reintervention because of new lesions or restenosis. In fracking procedures, additional sheath-site hemorrhages were observed, although no target lesion–specific complications were reported.^10^ Notably, several procedures reported zero acute complications at the lesion site, particularly when risk mitigation strategies were employed. One such strategy was the placement of distal embolic protection filters, especially in cases where distal embolization could not be confidently excluded.

## Discussion

### General findings

This review synthesized evidence from 15 studies involving 101 patients who underwent needle-based or plaque-modifying recanalization techniques for complex peripheral arterial lesions, particularly heavily calcified segments.^5^^,^^6^^,^^10^, ^11^, ^12^, ^13^, ^14^, ^15^, ^16^, ^17^, ^18^, ^19^, ^20^, ^21^, ^22^ Across studies, procedural success rates were consistently high, despite being performed in lesions resistant to conventional wire escalation.^5^^,^^6^^,^^10^, ^11^, ^12^, ^13^, ^14^, ^15^, ^16^, ^17^, ^18^, ^19^, ^20^, ^21^ Fracking and inner PIERCE demonstrated durable patency and low restenosis rates, with reported benefits extending from CFA interventions to BTK revascularization.^6^^,^^10^^,^^21^ Most techniques achieved rapid technical success without major complications; when present, adverse events were generally minor and manageable (eg, tract bleeding, nonflow-limiting dissections).^5^^,^^12^, ^13^, ^14^, ^15^, ^16^, ^17^^,^^20^, ^21^, ^22^ Several reports documented clinically meaningful improvements, including wound healing and limb salvage in chronic limb-threatening ischemia.^15^^,^^19^^,^^22^ The recently developed FRAP-CROSS technique further demonstrates that combining fracking with Rendezvous-PIERCE can enable guide wire passage even in long, densely calcified femoropopliteal occlusions that fail standard wiring and balloon escalation. Collectively, these findings highlight needle-based recanalization as a versatile option for challenging calcified lesions, adaptable to varied anatomic and procedural contexts.^5^^,^^6^^,^^10^, ^11^, ^12^, ^13^, ^14^, ^15^, ^16^, ^17^, ^18^, ^19^, ^20^, ^21^, ^22^ These approaches and their roles as escalation strategies are summarized in the Central Illustration.

**Central Illustration:**
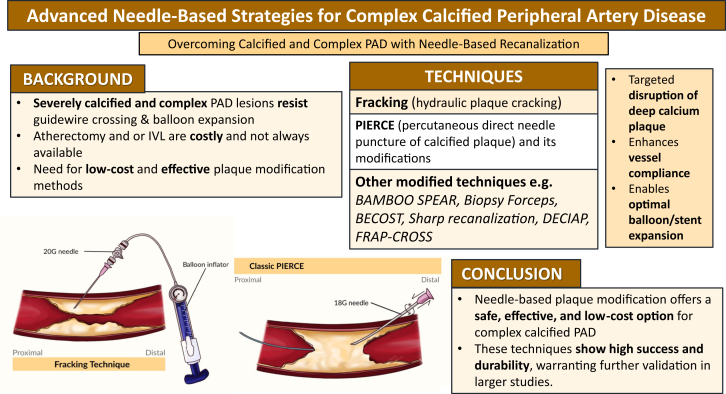
**Advancedneedle-basedstrategies for complex calcified peripheral artery disease (PAD).** Schematic overview of plaque-modifying needle-based techniques used for severely calcified PAD lesions refractory to conventional crossing or balloon dilation. BAMBOO SPEAR, needle drilling method; BECOST, blunt endoluminal cracking over strained through-wire; DECIAP, direct endarterectomy by clamp-induced artery plaque cracking; IVL, intravascular lithotripsy; PIERCE, percutaneous direct needle puncture of calcified plaque.

The heterogeneous distribution of target vessels highlights a practical advantage of needle-based techniques. Their flexibility across both large-caliber inflow arteries and small outflow or pedal vessels is especially relevant in chronic limb-threatening ischemia, where multilevel occlusions and distal targets may be involved. Despite the anatomic variance, high procedural success across vessel beds indicates that the underlying mechanical principles of controlled penetration and plaque modification remain broadly effective. However, the anatomic complexity of smaller or distal arteries necessitates heightened caution and tailored technique. Importantly, lesions amenable to these advanced approaches represent a clinically meaningful subset of peripheral arterial disease, typically involving focal, heavily calcified, or chronically occluded segments that are unsuitable for standard crossing techniques. Notably, lesions requiring these advanced approaches are not uncommon. Reports estimate that up to 40% of all peripheral interventions involve CTO with heavy calcification or failure of conventional crossing strategies.^23^ As such, these techniques represent not only salvage options but also important adjuncts in complex PAD management, provided they are performed by skilled operators with appropriate imaging support and institutional backup.

### Mechanism, advantages, and disadvantages of needle-based recanalization techniques and their modifications

Needle-based recanalization techniques are designed to overcome the mechanical barrier posed by heavily calcified lesions by creating a channel or enlarging the lumen through direct penetration or hydraulic disruption of plaque. These methods target specific anatomic and morphologic challenges that often prevent guide wire passage or adequate balloon expansion, particularly in focal lesions with concentric or eccentric heavy calcification, and especially when deep calcium is present.^4^^,^^24^

#### Fracking technique

The fracking technique employs a balloon-assisted hydraulic force delivered through a percutaneously inserted 18-G needle to fracture calcified plaque within the arterial wall. The procedure involves identifying a focal point of maximal calcium resistance under fluoroscopic or intravascular ultrasound (IVUS) guidance, advancing the needle into the plaque, and inflating the balloon until a sudden drop in inflation pressure indicates successful fracture—thereby increasing MLA and vessel compliance to facilitate subsequent balloon or stent expansion.^6^^,^^10^

Clinically, Haraguchi et al^5^ first demonstrated the feasibility of fracking in CFA lesions with marked IVUS-confirmed luminal expansion, absence of complications, and durable 2-year patency.^6^ In 2023, a comparative cohort confirmed fracking significantly outperformed balloon angioplasty in calcified CFA lesions—achieving higher procedural success, patency, and freedom from reintervention with a comparable safety profile; notably, a post-procedural MLA ≥16 mm^2^ emerged as a determinant of long-term durability.^10^ Ex vivo micro-CT further validated that fracking disrupts both superficial and deep nodular calcium, improving luminal geometry and flow characteristics.^25^ Despite these advantages, fracking demands millimeter-level targeting and meticulous pressure control to minimize perforation risk—limiting safe application to operators with advanced imaging skills and extensive experience.^6^^,^^10^^,^^24^

#### PIERCE technique and its modification

The PIERCE technique and its modifications use a needle to puncture the calcified cap/body to create a path for a guide wire or balloon passage. The technique is aimed at balloon-undilatable, focal, eccentric, and deeply calcified lesions, particularly when atherectomy devices cannot effectively reach or modify the target.^6^ The foundational study by Ichihashi et al^6^ showed that PIERCE enabled successful balloon passage and dilation in heavily calcified SFA/tibial lesions where standard ballooning failed—supporting its role as a bailout strategy for deep, resistant calcium.^6^

Modifications expand applicability: Rendezvous-PIERCE aligns antegrade and retrograde wires so that a retrograde needle can drill toward the antegrade wire without externalization^11^; inner PIERCE uses an externalized wire to guide precise needle advancement in small-caliber BTK arteries^20^; distal foot applications (eg, medial tarsal artery) have also been described.^21^ Clinical series of infrainguinal needle rendezvous report near-universal technical success with minimal complication rates in expert hands.^11^ Nonetheless, dual access, precise wire alignment, and coordinated team maneuvers add procedural complexity and a steep learning curve.^11^^,^^21^^,^^22^ A further evolution of this concept is the FRAP-CROSS technique, which integrates fracking with Rendezvous-PIERCE to achieve all-intracalcium wiring in femoropopliteal diffuse calcified occlusions. This technique allows a stentless strategy because hydraulic plaque fracture and needle-guided true-lumen wiring create a compliant, well-expanded lumen with minimal intimal injury, eliminating the dissections and recoil that would otherwise necessitate stenting.^12^

#### Other techniques

Other techniques leverage different physical principles. BECOST advances a blunt-tip needle over a through-and-through-wire to crack calcified segments—often BTK—with potentially lower perforation risk than sharp needles, albeit with longer procedural times.^15^ DECIAP is a hybrid surgical-endovascular approach where forceps inserted via a small incision crush the calcified wall under fluoroscopy, typically after failed intraluminal or subintimal attempts.^17^ Niche strategies include BAMBOO SPEAR (slightly curved 21-G needle to traverse calcified CFA cores),^12^ biopsy forceps extraction to physically debulk rigid plaque for optimal stent expansion,^13^ and sharp recanalization using the stiff end of a guide wire when conventional wiring fails.^16^^,^^17^ Notably, sharp recanalization and the inner PIERCE technique share the same core mechanistic features, relying on fluoroscopy-guided plaque penetration to enable guide wire passage. Inner PIERCE differs primarily in its intraluminal, wire-guided orientation, whereas sharp recanalization employs the stiff or sharp end of a guide wire. Nevertheless, both function as direct plaque penetration strategies rather than subintimal approaches, representing technical extensions of the same needle-assisted recanalization paradigm.

Across all these methods, the key advantage is immediate, controlled luminal entry/expansion in lesions previously deemed uncrossable, using widely available, relatively inexpensive tools—an asset in resource-limited settings.^1^^,^^12^, ^13^, ^14^, ^15^, ^16^, ^17^ They can also be deployed in device-inaccessible segments where atherectomy is impractical or intravascular lithotripsy is unavailable.^26^ However, success hinges on operator expertise, accurate lesion targeting, and robust procedural planning; centers should have advanced imaging, hybrid conversion capacity, and teams equipped to manage complications such as perforation, distal embolization, or acute vessel closure.^27^^,^^28^

### Future recommendation for daily practice

Needle-based recanalization should be incorporated as an escalation strategy for anatomically hostile lesions rather than as a first-line therapy—consistent with contemporary PAD guideline frameworks.^2^^,^^3^ Hybrid strategies such as FRAP-CROSS may be considered in selected cases where long, circumferential calcification prevents true-lumen crossing despite conventional intraluminal or subintimal techniques.^12^ These methods are most valuable for flush occlusions, reentry-resistant segments, and severe circumferential or nodular calcification where guide wire crossing, subintimal angioplasty, or reentry devices have failed.^4^

Successful adoption depends on operator skill, institutional readiness, and team coordination; procedures should be limited to centers with real-time IVUS/high-resolution fluoroscopy and immediate hybrid surgical backup.^6^ Post-procedural follow-up should standardize assessments of luminal gain, stent expansion, and restenosis. Integration with vessel-preparation strategies—high-pressure balloons, drug-coated balloons, or intravascular lithotripsy—may optimize long-term outcomes in selected anatomies.^4^^,^^28^ Finally, broader use requires multicenter registries and prospective studies with standardized outcome definitions, enabling comparisons among needle-based strategies and informing future guideline statements.^5^^,^^6^

### Strengths and limitations

A key strength of this review is its comprehensive scope, encompassing both widely practiced and niche needle-based recanalization techniques across multiple peripheral arterial territories. By integrating case reports, case series, and cohort studies, this analysis preserves critical procedural details—such as lesion morphology, device specifications, and access strategies—that are often omitted in larger data sets. The inclusion of diverse anatomic targets and patient populations also reflects the adaptability of these methods across real-world scenarios, from primary interventions to last-resort salvage techniques. However, the available evidence has inherent limitations. Although many techniques are described, most of the reported cases come from a very limited number of publications, which restricts the strength and generalizability of the evidence. In addition, most reports were also small, observational, and noncomparative. Reporting standards varied widely, with inconsistent definitions for technical success, patency, and complications, making meta-analysis infeasible. Follow-up durations were often short to midterm, potentially underestimating late restenosis or reintervention rates. Furthermore, publication bias is likely, as highly selected, technically successful cases may be overrepresented, and the procedural expertise required for many of these techniques may not be readily transferable to all practice environments. To address these limitations, future multicenter prospective studies with standardized outcome definitions and longer follow-up are needed to establish evidence-based guidelines for the optimal use of various needle-based recanalization techniques in complex PAD.

## Conclusion

Needle-based and plaque-modifying interventions, such as fracking, PIERCE, and its modifications, were reported to deliver high technical success rates, substantial luminal gain, and sustained vessel patency in complex and/or heavily calcified lesions resistant to conventional endovascular therapy. These techniques provide precise, mechanically targeted plaque disruption, enabling improved vessel compliance and optimal stent deployment, with complications being uncommon and generally manageable. Their applicability across diverse vascular territories and reliance on readily available tools make them valuable additions to the interventional repertoire, particularly in anatomically hostile lesions. Emerging hybrid approaches, including inner PIERCE and FRAP-CROSS, further expand applicability by combining hydraulic, mechanical, and needle-guided recanalization principles to achieve true-lumen crossing in lesions previously deemed uncrossable. However, these procedures require advanced technical skill, precise imaging guidance, and coordinated team execution, and should therefore be performed only by experienced operators in specialized centers equipped to manage potential complications. Future progress will depend on well-designed multicenter trials with standardized protocols and extended follow-up to validate long-term benefits, optimize patient selection, and formalize their role within evidence-based treatment algorithms for complex PAD.

## Footnotes

To access the supplementary material accompanying this article, visit the online version of the *Journal of the Society for Cardiovascular Angiography & Interventions* at 10.1016/j.jscai.2026.105306.
