# Supplementary material for: Advanced Needle-Based Strategies for Complex Calcified Peripheral Artery Disease: A Systematic Review
Source: J Soc Cardiovasc Angiogr Interv. 2026 Apr 9;5(5):105306. doi: 10.1016/j.jscai.2026.105306 (PMC13198127; doi:10.1016/j.jscai.2026.105306)
Supplement: Supplementary Material [file mmc1.docx]

**From Lesion Engagement to Plaque Modification: Advanced Needle-Based Strategies for**

**Complex Calcified Peripheral Artery Disease**

**Supplementary Materials**

**Supplemental Table S1.** PRISMA 2020 Checklist

**Supplemental Figure S1.** PRISMA flow diagram of the study selection process

**Supplemental Table S2.** Baseline Characteristics of Included Studies

**Supplemental Table S3.** Lesion, Procedural Characteristics and Outcomes of Included Studies

**Supplemental Tables S4 and S5.** Risk of Bias Assessment of Included Studies

**Supplemental Table S1.** PRISMA 2020 Checklist

| **Topic** | **No.** | **Item** | **Location where item is reported** |
| --- | --- | --- | --- |
| **TITLE** |  |  |  |
| **Title** | **1** | **Identify the report as a systematic review.** | **1** |
| **ABSTRACT** |  |  |  |
| **Abstract** | **2** | **See the PRISMA 2020 for Abstracts checklist** |  |
| **INTRODUCTION** |  |  |  |
| **Rationale** | **3** | **Describe the rationale for the review in the context of existing knowledge.** | **1-3** |
| **Objectives** | **4** | **Provide an explicit statement of the objective(s) or question(s) the review addresses.** | **1-3** |
| **METHODS** |  |  |  |
| **Eligibility criteria** | **5** | **Specify the inclusion and exclusion criteria for the review and how studies were grouped for the syntheses.** | **2-4** |
| **Information sources** | **6** | **Specify all databases, registers, websites, organisations, reference lists and other sources searched or consulted to identify studies. Specify the date when each source was last searched or consulted.** | **2-4** |
| **Search strategy** | **7** | **Present the full search strategies for all databases, registers and websites, including any filters and limits used.** | **2-4** |
| **Selection process** | **8** | **Specify the methods used to decide whether a study met the inclusion criteria of the review, including how many reviewers screened each record and each report retrieved, whether they worked independently, and if applicable, details of automation tools used in the process.** | **2-4** |
| **Data collection process** | **9** | **Specify the methods used to collect data from reports, including how many reviewers collected data from each report, whether they worked independently, any processes for obtaining or confirming data from study investigators, and if applicable, details of automation tools used in the process.** | **2-4** |
| **Data items** | **10a** | **List and define all outcomes for which data were sought. Specify whether all results that were compatible with each outcome domain in each study were sought (e.g. for all measures, time points, analyses), and if not, the methods used to decide which results to collect.** | **2-4** |
|  | **10b** | **List and define all other variables for which data were sought (e.g. participant and intervention characteristics, funding sources). Describe any assumptions made about any missing or unclear information.** | **2-4** |
| **Study risk of bias assessment** | **11** | **Specify the methods used to assess risk of bias in the included studies, including details of the tool(s) used, how many reviewers assessed each study and whether they worked independently, and if applicable, details of automation tools used in the process.** | **2-4** |
| **Effect measures** | **12** | **Specify for each outcome the effect measure(s) (e.g. risk ratio, mean difference) used in the synthesis or presentation of results.** | **2-4** |
| **Synthesis methods** | **13a** | **Describe the processes used to decide which studies were eligible for each synthesis (e.g. tabulating the study intervention characteristics and comparing against the planned groups for each synthesis (item 5)).** | **2-4** |
|  | **13b** | **Describe any methods required to prepare the data for presentation or synthesis, such as handling of missing summary statistics, or data conversions.** | **2-4** |
|  | **13c** | **Describe any methods used to tabulate or visually display results of individual studies and syntheses.** | **2-4** |
|  | **13d** | **Describe any methods used to synthesize results and provide a rationale for the choice(s). If meta-analysis was performed, describe the model(s), method(s) to identify the presence and extent of statistical heterogeneity, and software package(s) used.** | **2-4** |
|  | **13e** | **Describe any methods used to explore possible causes of heterogeneity among study results (e.g. subgroup analysis, meta-regression).** | **2-4** |
|  | **13f** | **Describe any sensitivity analyses conducted to assess robustness of the synthesized results.** | **2-4** |
| **Reporting bias assessment** | **14** | **Describe any methods used to assess risk of bias due to missing results in a synthesis (arising from reporting biases).** | **2-4** |
| **Certainty assessment** | **15** | **Describe any methods used to assess certainty (or confidence) in the body of evidence for an outcome.** | **2-4** |
| **RESULTS** |  |  |  |
| **Study selection** | **16a** | **Describe the results of the search and selection process, from the number of records identified in the search to the number of studies included in the review, ideally using a flow diagram.** | **4-9** |
|  | **16b** | **Cite studies that might appear to meet the inclusion criteria, but which were excluded, and explain why they were excluded.** | **4-9** |
| **Study characteristics** | **17** | **Cite each included study and present its characteristics.** | **4-9** |
| **Risk of bias in studies** | **18** | **Present assessments of risk of bias for each included study.** | **4-9** |
| **Results of individual studies** | **19** | **For all outcomes, present, for each study: (a) summary statistics for each group (where appropriate) and (b) an effect estimate and its precision (e.g. confidence/credible interval), ideally using structured tables or plots.** | **4-9** |
| **Results of syntheses** | **20a** | **For each synthesis, briefly summarise the characteristics and risk of bias among contributing studies.** | **4-9** |
|  | **20b** | **Present results of all statistical syntheses conducted. If meta-analysis was done, present for each the summary estimate and its precision (e.g. confidence/credible interval) and measures of statistical heterogeneity. If comparing groups, describe the direction of the effect.** | **4-9** |
|  | **20c** | **Present results of all investigations of possible causes of heterogeneity among study results.** | **4-9** |
|  | **20d** | **Present results of all sensitivity analyses conducted to assess the robustness of the synthesized results.** | **4-9** |
| **Reporting biases** | **21** | **Present assessments of risk of bias due to missing results (arising from reporting biases) for each synthesis assessed.** | **4-9** |
| **Certainty of evidence** | **22** | **Present assessments of certainty (or confidence) in the body of evidence for each outcome assessed.** | **4-9** |
| **DISCUSSION** |  |  |  |
| **Discussion** | **23a** | **Provide a general interpretation of the results in the context of other evidence.** | **10-12** |
|  | **23b** | **Discuss any limitations of the evidence included in the review.** | **10-12** |
|  | **23c** | **Discuss any limitations of the review processes used.** | **10-12** |
|  | **23d** | **Discuss implications of the results for practice, policy, and future research.** | **10-12** |
| **OTHER INFORMATION** |  |  |  |
| **Registration and protocol** | **24a** | **Provide registration information for the review, including register name and registration number, or state that the review was not registered.** | **2-3** |
|  | **24b** | **Indicate where the review protocol can be accessed, or state that a protocol was not prepared.** | **2-3** |
|  | **24c** | **Describe and explain any amendments to information provided at registration or in the protocol.** | **2-3** |
| **Support** | **25** | **Describe sources of financial or non-financial support for the review, and the role of the funders or sponsors in the review.** | **14-15** |
| **Competing interests** | **26** | **Declare any competing interests of review authors.** | **14-15** |
| **Availability of data, code and other materials** | **27** | **Report which of the following are publicly available and where they can be found: template data collection forms; data extracted from included studies; data used for all analyses; analytic code; any other materials used in the review.** | **14-15** |

**Supplemental Figure S1.** PRISMA flow diagram of the study selection process

**Supplemental Table S2.** Baseline Characteristics of Included Studies

| ***No.*** | ***Author, Year*** | ***Study Design*** | ***Study Location*** | ***Sample (n)*** | ***Male, n(%)*** | ***Age*** | ***BMI*** | ***Comorbidity*** | | | | | |
| --- | --- | --- | --- | --- | --- | --- | --- | --- | --- | --- | --- | --- | --- |
|  |  |  |  |  |  |  |  | **Hypertension** | **Diabetes Mellitus** | **Dyslipidemia** | **Smoking** | **CAD** | **CKD** |
| 1 | Haraguchi et al., 2021a  (Fracking) | Case report | Japan | 2 | 2 (100%) | 72-81 | N/A | N/A | 1 (50%) | N/A | N/A | N/A | 1 (50%) |
| 2 | Haraguchi et al., 2023  (Fracking vs POBA) | Retrospective cohort | Japan | 59 | 38(64%) | 77.0 ± 8.0 | Fracking: 21.3 ± 2.9  Ballon: 22.8 ± 4.2 | Fracking: 25 (83%)  Balloon: 27 (93%) | Fracking: 17 (57%)  Balloon: 22 (76%) | Fracking: 21 (70%)  Balloon: 22 (76%) | Fracking: 6 (20%)  Balloon: 6 (21%) | Fracking: 20 (67%)  Balloon: 24 (83%) | Fracking:20 (67%)  Balloon: 20 (69%) |
| 3 | Haraguchi et al., 2024  (Rendezvous-PIERCE) | Case series | Japan | 2 | 2 (100%) | 68-80 | N/A | N/A | N/A | N/A | N/A | N/A | N/A |
| 4 | Haraguchi et al., 2025  (FRAP-CROSS) | Case report | Japan | 1 | 1 (100%) | 90 | N/A | N/A | N/A | N/A | N/A | N/A | N/A |
| 5 | Hayakawa et al., 2021  (BAMBOO SPEAR) | Case report | Japan | 1 | 1 (100%) | 73 | N/A | N/A | 1 (100%) | N/A | N/A | 1 (100%) | 1 (100%) |
| 6 | Hirano et al., 2021  (PIERCE) | Case report | Japan | 1 | 1 (100%) | 78 | N/A | N/A | 1 (100%) | N/A | N/A | N/A | N/A |
| 7 | Horsirimanont et al., 2025  (PIERCE) | Case report | Thailand | 5 | 4 (80%) | 59-89 | N/A | N/A | 4 (80%) | N/A | N/A | N/A | 2 (40%) |
| 8 | Huang et al., 2012  (CR SHARP) | Case report | China | 2 | 0 (0%) | 67-82 | N/A | 1 (50%) | 1 (50%) | N/A | N/A | 1 (50%) | 1 (50%) |
| 9 | Ichihashi et al., 2014  (PIERCE) | Case series | Japan | 4 | 3 (75%) | 55-85 | N/A | N/A | 4 (100%) | N/A | N/A | 2 (50%) |  |
| 10 | Kim et al., 2020  (PIERCE) | Case series | United Kingdom and United Arab Emirates | 2 | 2 (100%) | 65-73 | N/A | N/A | N/A | N/A | N/A | N/A | N/A |
| 11 | Kum et al., 2020  (PIERCE) | Case series | Singapore | 4 | 1 (25%) | 73-83 | 21.5-28.5 | 4 (100%) | 4 (100%) | 4 (100%) | N/A | 1 (25%) | 1 (25%) |
| 12 | Nakama et al., 2020  (PIERCE) | Case series | Japan | 1 | 1 (100%) | 65 | N/A | N/A | N/A | N/A | N/A | 1 (100%); Post CABG | N/A |
| 13 | Takamura et al., 2016 | Case report | Japan | 1 | 1 (100%) | Early 70s | N/A | N/A | 1 (100%) | N/A | N/A | N/A | 1 (100%); Post transplant |
| 14 | Takei et al., 2021  (PIERCE) | Retrospective cohort | Japan | 15 | 14 (93.3%) | 73.4±7.6 | N/A | 11 (73.3%) | 8 (53.3%) | 7 (46.7%) | 10 (66.7%) | 7 (46.7%) | 14 (93.3%) |
| 15 | Troisi et al., 2023  (CR PIERCE) | Case report | Italy | 1 | 0 (0%) | 82 | N/A | N/A | N/A | N/A | N/A | N/A | N/A |

| **Supplemental Table S3.** Lesion, Procedural Characteristics and Outcomes of Included Studies | | | | | | | | | | | | | | |
| --- | --- | --- | --- | --- | --- | --- | --- | --- | --- | --- | --- | --- | --- | --- |
| **No** | **Author, Year** | ***Lesion Characteristics*** | | | | | | ***Procedural characteristics*** | | | | | ***Follow-up duration*** | **Outcomes** |
|  |  | **Baseline ABI** | **Baseline stenosis (%)** | ***Minimal Lumen Area*** | **Lesion Location** | **Plaque Characteristics** | **Technique** | **Description** | **Pros** | **Cons** | ***Utilized Devices*** | **Procedure Time** |  |  |
| 1 | Haraguchi et al., 2021a | N/A | 94-96% | - Case 1: 6.2 mm2 (3.9 × 2.2-mm)  - Case 2: 10.0 mm2 (7.0 × 40-mm) | CFA | Severely eccentric calcified plaque in mid-distal CFA | Fracking | Hydraulic pressure via 18G needle cracks calcium during balloon inflation until pressure drop. | Safe; improves MLA significantly; no device shift | Limited use cases reported; requires precise pressure control | 18 G needle (Terumo, Japan) without a plastic outer sheath | N/A | 2 years | MLA ↑ from ~7 to >27 mm²; 0% Residual stenosis; Long-term patency >24 months; No complications |
| 2 | Haraguchi et al., 2023 | ABI improvement  Fracking: 0.99±0.20  Balloon: 0.78±0.28 | Residual stenosis  Fracking: 19.5±12.3%  Balloon: 28.6±19.3% | Baseline  Fracking: 5.7±3.1 ­mm2  Balloon: 5.9±3.7 ­mm2  Post-procedural  Fracking: 22.1±4.0 ­mm2  Balloon: 12.7±3.3 ­mm2  Acute luminal gain  Fracking: 16.4±4.8 ­mm2  Balloon:  6.8±4.4 ­mm2 | CFA | Severe calcification in 180–360°  Fracking: 81.3%  Balloon: 48.6%  CTO  Fracking: 4 (12%)  Balloon: 7 (20%)  Lesion length:  Fracking: 27.5±12.6  Balloon: 31.4±6.9 | Fracking vs Balloon | Fracking compared with balloon PTA in calcified lesions. | Higher success and patency; safe profile | Requires pressure control; IVUS needed for MLA | 18 G needle (Terumo, Japan) without a plastic outer sheath | 10.1±3.8 minutes.  Radiation time  Fracking: 25.5±13.8 minutes  Balloon: 51.6±44.3 minutes | Fracking: 432±222 days  Balloo: 377±248 days | **Procedural success:**  Fracking: 31 (96.9%)  Balloon: 26 (74.3%);  **Primary Patency:**  Fracking: 29 (89.8%)  Balloon: 19 (49.2%);  **Restenosis:**  Fracking: 3 (9.4%)  Balloon: 15 (42.8%);  **Reocclusion:**  Fracking: 0 (0%)  Balloon: 2 (5.7%);  **Freedom from TLR:**  Fracking: 30 (93.5%)  Balloon: 27 (74.2%);  **Freedom from MALE:**  Fracking: 16 (76.9%)  Balloon: 13 (48.6%);  **Residual stenosis:**  Fracking: 19.5±12.3%  Balloon: 28.6±19.3%. |
| 3 | Haraguchi et al., 2024 | N/A | N/A | N/A | Case 1: ATA  Case 2: Femoropopliteal | Case 1: Calcified lesion in proximal ATA  Case 2: Calcified lesion | Rendezvous PIERCE | Retrograde needle targets antegrade wire; drills calcium without externalisation. | Effective when wires fail; extends PIERCE applicability | Requires dual access and high fluoroscopy skill | 18- or 20- G needle (with outer diameters of 1.3 mm and 0.9 mm, respectively) | N/A | 6 months | Balloon crossed; Hemostasis successful; No complications; Asymptomatic at 6–12 mo; no re-intervention |
| 4 | Haraguchi et al., 2025 | N/A | N/A | N/A | Femoropopliteal | Diffused calcified occlusions | FRAP-CROSS | Hybrid technique combining Fracking and Rendezvous-PIERCE to achieve intracalcium guidewire passage through long, heavily calcified femoropopliteal occlusions. | Enables wiring; maintains an intraluminal trajectory; supports stentless treatment; requires only standard needles and balloons. | Multiple punctures increase technical complexity; requires precise antegrade-retrograde alignment; limited evidence and unknown long-term durability. | 20-G metal needle without  the plastic outer sheath from an 18-G needle (Terumo, Japan) or a 21-G metal needle (micro-puncture introducer set, Cook Medical, USA) | N/A | N/A | Successful guidewire advancement and subsequent balloon angioplasty with no perforation, no distal embolization, and no flow-limiting dissection reported |
| 5 | Hayakawa et al., 2021 | N/A | N/A | N/A | CFA | Severe total occlusion in CFA with calcified plaque | BAMBOO SPEAR | 21G needle punctures core of plaque under angio-guidance; wire advanced through hollow. | Precise; no atherectomy needed | Single report; technically demanding | 21 G metal needle that was slightly curved into the blood vessel from where the lumen of the distal CFA was located | N/A | 5 months | Wire passed centrally; effective DCB; No complications; Vessel patency at 5-month angiographic follow-up and IVUS also showed sufficient lumen area |
| 6 | Hirano et al., 2021 | 0.77 |  |  | SFA | CTO in proximal to mid SFA, with severe calcified plaque at ostium SFA | Biopsy Forceps Extraction | Biopsy forceps remove calcified plaque repeatedly to create path for stent. Manuever were repeated 21 times. | Complete calcium removal | Off-label; requires expertise | Biopsy forceps (Technowood, Tokyo, Japan) | N/A | 9 months | Full stent expansion; no dissection or DFA shift; No restenosis or complications; At the follow-up, patient remain symptoms free with normalised ABI at 1.06 |
| 7 | Horsirimanont et al., 2025 | N/A | N/A | N/A | BTK (ATA, PTA, LPA) | Heavily calcified lesion +/- CTO  Occlusion: 4 (80%)  Stenosis: 1 (20%) | BECOST (Blunt needle over wire) | Blunt needle Endoluminal Cracking Over Strained Through-and-through wire. Used over a through-wire to crack calcified BTK plaque. | Safe over strained wire; effective after BADFORM failure; avoids sharp penetration. | Requires through-and-through access; limited to BTK; not suitable in all anatomy. | Blunt-tip needle, with length determined by the distance from the distal puncture site or site of wire exteriorization to the proximal end of the stuck lesion. | 230-510 minutes | 3 months | Technical Success Rate: 100%; No guidewire Issues; No vessel rupture; All lesions successfully dilated with high-pressure balloon  Dissections: 3 (75%) cases of minimal, non-flow-limiting dissection  Stenting: 1 (25%) case required a drug-eluting stent  Puncture Site Complications: 1 (25%) case required balloon-assisted thrombin injection  Wound Healing:  - 3 (75%) cases had complete healing within 3 months after minor amputation  - 1 (25%) case had successful foot loop recanalization, but no outflow  Reintervention: 1 (25%) case due to restenosis within 3 months |
| 8 | Huang et al., 2012 | 0.35-0.45 | N/A | N/A | SFA, popliteal | Heavily calcified occlusion | Sharp recanalization (stiff guidewire) | Stiff end of guidewire used to pierce calcified cap when conventional wire fails. | Simple; accessible tools; no special equipment. | Risk of dissection/perforation; limited control. | Terumo stiff glidewire (Radifocus, Terumo, Tokyo, Japan), supported with 5-Fr right Judkins (JR) catheter | N/A | 3-7 months | Both successfully crossed and stented after conventional failure; Post-procedural ABI improvement  Case 1:  - ABI increased to 0.78  - Second toe amputated 1 month post-EVI  - Reintervention at 7 months for Left CIA lesion and In-stent restenosis in SFA  Case 2:  - No distal embolism  - ABI increased to 0.94 within 1 week  - No ischemic symptoms or complications within 3 months |
| 9 | Ichihashi et al., 2014 | N/A | N/A | N/A | SFA: 3 (75%); PTA: 1 (25%) | Heavily calcified occlusion | PIERCE (needle puncture) | Direct needle puncture through calcified plaque under fluoroscopy to enable balloon crossing. | Enables balloon crossing; inexpensive; simple. | Bleeding risk (2 cases); requires precise targeting. | 19-gauge needle (HAKKO ELASTER; Hakko, Nagano, Japan) in cases 1 and 2 and a 16-gauge needle in cases 3 and 4 under  fluoroscopic guidance. | N/A | N/A | 100% balloon passage; minor bleeding from punctured tract in 2 (50%) cases; Stent placement in SFA occlusions (n=3; 75%) due to residual stenosis; No distal embolization and No other perioperative adverse events |
| 10 | Kim et al., 2020 | N/A | N/A | N/A | CIA, ATA | Chronic total occlusion | Sharp recanalization | Same as above but in CIA and ATA. | Useful for calcified caps; commonly available tools. | Risk of perforation; not ideal in tortuous vessels. | N/A | N/A | N/A | Success in both after conventional failed |
| 11 | Kum et al., 2020 | N/A | 70-100% | N/A | ATA: 3 (75%)  SFA: 1 (25%);  CLTI Rutherford Class 5–6 | Diffuse stenosis and occlusion with moderate-severe calcification (PARC Classification of Calcificiation); TASC C-D | DECIAP (artery forceps cracking) | External artery forceps crack calcified segment after wire/PIERCE fail. | Effective when all else fails; external technique. | Requires incision; potential tissue damage. | 16 G needle | N/A | 3 months | - Balloon success after PIERCE + DECIAP in all cases  - Residual stenosis: 0% in 2 patients (no residual stenosis); ≤30% in 2 patients (angiographically insignificant)  - No distal embolization and aneurysmal changes  - Complication: 1 cases of small arterial perforation at DECIAP site, and resolved with balloon; 1 patient died of sepsis within 6 days post-procedure  - 1 case of clinically driven reintervention due to occlusion at 3 months  - Wound healed at 6-131 days |
| 12 | Nakama et al., 2020 | N/A | N/A | N/A | PTA, LPA | Multiple severe calcifications in PA and PTA; Severely calcified and occluded distal PTA and LPA | Inner PIERCE (needle over wire) | Needle inserted through externalized retrograde wire to cross calcified BTK lesion. | Precise; leverages retrograde support. | Needs bidirectional access and externalized wire. | Percutaneous trans-hepatic cholangiodrainage (PTCD) needle (; Happy-cath PTCD needle; Medikit, Tokyo, Japan). | N/A | 1 year | Angioplasty successful; No resetnosis and complication at 3-month aniography follow-up; Wound healed in 4 months; No woound recurrence was observed after 1 year |
| 13 | Takamura et al., 2016 | 0.45 | N/A | N/A | SFA | Calcified plaque and CTO in distal SFA | PIERCE | Same method applied to SFA CTO for balloon passage. | Enables balloon/stent delivery in long CTOs. | Operator-dependent; bleeding risk. | 21-gauge needle (HAKKO  ELASTER, Hakko, Japan), | N/A | N/A | Enabled balloon, stent deployed with optimal result |
| 14 | Takei et al., 2021 | N/A | N/A | N/A | ATA: 4 (22.2%)  PTA: 10 (55.6%)  Dorsal artery: 4 (22.2%) | Severe calcification: 18/18 (100%)  CTO: 15/18 (83.3%)  Stenosis: 3/18 (16.7%) | Inner PIERCE | Needle guided through externalized wire to enable ballooning of calcified BTA. | High success (94.4%); no complications. | Complex setup; requires operator skill. | 20 G, 105 mm long puncture needle (MEDIKIT) OR the 20 G, 310 mm long biopsy needle (NIPRO) | N/A | N/A | Technical success: 94.4% with <30% residual stenosis; 100% successful device delivery with sufficient dilatation; No complications |
| 15 | Troisi et al., 2023 | N/A | N/A | N/A | Medial tarsal artery (BTA) | High-grade calcification in medial tarsal artery | Modified PIERCE | Radial cannulation needle cracks calcified medial tarsal artery for angiosome-targeted therapy. | Allows distal revascularization in BTA; effective. | Small target; technical challenge; off-label tool. | 20 G radial cannulation needle | N/A | 10 days | Direct flow restored; DUS showed patency with PSV 71 cm/s and TBI 0.92; Discharged within 10 days with almost healed wound. |

**Supplemental Tables S4 and S5.** Risk of Bias Assessment of Included Studies

| **Newcastle–Ottawa Scale (NOS)** | | | | | | | | |
| --- | --- | --- | --- | --- | --- | --- | --- | --- |
| **No** | **Author, Year** | ***Study Design*** | **Selection** | **Selection** | **Comparability** | **Outcome** | **Total** | **Category** |
| 1 | Haraguchi et al., 2023 | Retrospective cohort | Newcastle–Ottawa Scale (NOS) | 4 | 2 | 3 | 9 | Low Risk |
| 2 | Takei et al., 2021 | Retrospective cohort | Newcastle–Ottawa Scale (NOS) | 3 | 2 | 3 | 8 | Low Risk |

| **Joanna Briggs Institute (JBI) scale quality assessment** | | | | | | | | | | | | |
| --- | --- | --- | --- | --- | --- | --- | --- | --- | --- | --- | --- | --- |
| **No** | **Author, Year** | ***Study Design*** | **Q1** | **Q2** | **Q3** | **Q4** | **Q5** | **Q6** | **Q7** | **Q8** | **Q9** | **Q10** |
| 1 | Haraguchi et al., 2021a | Case report | Y | Y | Y | Y | Y | Y | Y | Y | Y | N/A |
| 2 | Haraguchi et al., 2024 | Case series | Y | Y | Y | Y | Y | Y | Y | Y | Y | N/A |
| 3 | Haraguchi et al., 2025 | Case report | Y | Y | Y | Y | Y | Y | Y | Y | Y | N/A |
| 4 | Hayakawa et al., 2021 | Case report | Y | Y | Y | Y | Y | Y | Y | Y | Y | N/A |
| 5 | Hirano et al., 2021 | Case report | Y | Y | Y | Y | Y | Y | Y | Y | Y | N/A |
| 6 | Horsirimanont et al., 2025 | Case report | Y | Y | Y | Y | Y | Y | Y | Y | Y | N/A |
| 7 | Huang et al., 2012 | Case report | Y | Y | Y | Y | Y | Y | Y | Y | Y | N/A |
| 8 | Ichihashi et al., 2014 | Case series | Y | Y | Y | Y | Y | Y | Y | N | Y | N/A |
| 9 | Kim et al., 2020 | Case series | Y | Y | Y | Y | Y | Y | Y | N | Y | N/A |
| 10 | Kum et al., 2020 | Case series | Y | Y | Y | Y | Y | Y | Y | Y | Y | N/A |
| 11 | Nakama et al., 2020 | Case series | Y | Y | Y | Y | Y | Y | Y | Y | Y | N/A |
| 12 | Takamura et al., 2016 | Case report | Y | Y | Y | Y | Y | Y | Y | N | Y | N/A |
| 13 | Troisi et al., 2023 | Case report | Y | Y | Y | Y | Y | Y | Y | Y | Y | N/A |
